# Getting the right dose in cancer chemotherapy – time to stop using surface area?

**DOI:** 10.1038/sj.bjc.6600226

**Published:** 2002-04-22

**Authors:** D R Newell

**Affiliations:** Cancer Research UK Developmental Therapeutics Programme, Medical School, University of Newcastle, Newcastle NE2 4HH, UK

## Abstract

*British Journal of Cancer* (2002) **86**, 1207–1208. DOI: 10.1038/sj/bjc/6600226 www.bjcancer.com

© 2002 Cancer Research UK

## 

The successful treatment of cancer using chemotherapy requires two conditions to be met. For each patient, and their tumour, the correct drug(s) must be selected and the right dose(s) must be administered. Pre-clinical and clinical research in cancer chemotherapy is currently focused on satisfying the first condition, mainly through the search for new drugs with greater activity and tumour selectivity. The generic approach being taken is to identify targets related to the molecular or cellular pathology of cancer, followed by the development of agents which interact with these targets and produce the required change in tumour cell biology or host-tumour interactions. Target-based drug development is beginning to bare fruit and there is widespread optimism that a new generation of more selective and more active drugs will soon be with us.

The paper by Gurney in the current issue (Gurney, 2002) addresses the second of the two conditions that must be met for successful treatment, namely, the selection of the correct drug dose for each patient. The convention for many decades has been to use body surface area to calculate drug doses, the rationale being that drug clearance varies as a function of this parameter reflecting relationships between surface area and metabolic rate, blood flow, renal function, etc. Thus, patients receive a fixed number of, for example, mg per m^2^, and a number of nomograms and equations exist for calculating surface area from weight and height. The accuracy of these methods of calculating surface area has been the subject of much debate, and this calculation alone introduces inaccuracies. On top of these inaccuracies, and of greater importance, there are significant inter-patient, and to a lesser extent intra-individual, differences in drug clearance that are not related to surface area. Gurney lists a number of specific reasons for surface area-independent variations in drug clearance, and these include variability in the activity of drug metabolising enzymes and transporters for genetic reasons, pharmacokinetic interactions due to concomitant medication, and impaired organ function due to either disease or prior therapy. Given all these very real issues, and the compelling case made by Gurney, it is amazing that the habit of using surface area to adjust doses has persisted for so long. A major reason for using surface area in the first instance stems from the early data of Freireich and colleagues (Freireich *et al*, 1966), who showed that maximum tolerated doses of cytotoxic drugs where often the same in different species, ranging from mice to humans, when doses were scaled according to surface area. Toxic doses were less consistent when body weight was used to normalise doses. The consistency of toxic doses across species when normalised to body surface area reflects the general relationship between surface area and a number of physiological processes which together determine drug clearance, as alluded to above. There is of course no doubt that drug doses in children, particularly infants, must be reduced relative to doses in adults. However, in an adult population there is really no reason to use surface area in determining doses, unless surface area does correlate with clearance across the range of body sizes seen in the target adult population.

Table 4 in Gurney's paper provides 12 commonsense ‘rules’ that every physician should have in mind when prescribing doses of cytotoxic therapy, and the role of pharmacists and nurses in this process is similarly important. Taking account of these issues should ensure that a safe, but not sub-therapeutic, dose is prescribed on the first occasion a patient is treated. Subsequently, biological endpoints (rule 11) should be used to adjust doses for each individual patient, as previously advocated by the author (Gurney, 1996), to ensure that over the course of therapy each individual patient is given the best chance of therapeutic benefit whilst being exposed to manageable and not excessive levels of toxicity. In the case of cytotoxic drugs, the biological endpoint most frequently used as a surrogate for antiproliferative activity against tumour cells is damage to diving normal tissues, and myelosuppression and mucosistis are frequently used to adjust doses. Unfortunately, as noted by Gurney, these adjustments rarely include dose escalation in patients in whom no toxicity is observed, with the result that such paitents are likely to be under treated. Whilst the approach proposed by Gurney is eminently sensible for cytotoxic drugs, which have a narrow therapeutic index and a sensitive normal tissue where toxicity is related to the mechanism of drug action and easy to measure, the same may not be true for the new generation of cancer drugs. For more selective targeted drugs, the therapeutic index may be wider and fixed dosing, i.e. not adjusted on the basis of surface area, has already been successfully used in a number of trials, particularly where the drug is given orally. The real challenge is to find ways of monitoring the biological activity of these new treatments in patients to ensure that individuals are given potentially active doses, and non-invasive pharmacodynamic techniques (e.g. functional imaging) may have a role in this respect.

An alternative to measuring biological endpoints for dose optimisation is to use pharmacokinetic data, often referred to as therapeutic drug monitoring. This approach is applicable to any drug for which target plasma concentrations have been identified, and thus it applies to both cytotoxic and non-cytotoxic therapies. Evans and colleagues have shown that in childhood acute lymphoblastic leukaemia dose adjustment on the basis of pharmacokinetic data can indeed result in improved outcome results (Evans *et al*, 1998), although the logistical challenges involved in delivering pharmacokinetically-guided dosing can be substantial.

Gurney's paper comes as a timely reminder that for maximum efficacy dose can be as important as selecting the right drug, and that optimum doses for individual patients will vary widely. The approach advocated by Gurney, namely to take all possible pre-treatment and post-treatment clinical and pharmacological data into account in prescribing, makes eminent sense. In particular, reliance upon surface area as the primary determinant of dose should be seriously questioned. For new agents, Phase II and Phase III trials in adults should avoid using surface area-normalised dosing. An exception would be when pharmacokinetic data show that drug clearance is significantly related to body surface area, and that an alternative simple physiological parameter, e.g. renal function, is not more significantly related. For established drugs, a widespread move away from surface area based dosing is likely to need randomised trials to confirm that so doing does not impair efficacy or result in increased toxicity. Unfortunately, to be sure that the incidence of rare by serious toxicities is not increased, such trials would need to be large and realistically they are unlikely to be performed. Nevertheless, for standard patient care, the rules defined by Gurney are an excellent step in the right direction for physicians, pharmacists and nurses who are prepared to get to grips with the pharmacology of the drugs they use.
